# Proportion of Pre-Symptomatic Transmission Events Associated with COVID-19 in South Korea

**DOI:** 10.3390/jcm11143925

**Published:** 2022-07-06

**Authors:** Youngji Song, Eunha Shim

**Affiliations:** Department of Mathematics, Soongsil University, Seoul 06978, Korea; syj7825@soongsil.ac.kr

**Keywords:** COVID-19, Korea, pre-symptomatic, serial interval, incubation period, SARS-CoV-2, statistical, mathematical, expanded testing, Delta variant

## Abstract

Pre-symptomatic transmission potentially reduces the effectiveness of symptom-onset-based containment and control strategies for the coronavirus disease (COVID-19). Despite evidence from multiple settings, the proportion of pre-symptomatic transmission varies among countries. To estimate the extent of pre-symptomatic transmission in South Korea, we used individual-level COVID-19 case records from the Korea Disease Control and Prevention Agency and Central Disease Control Headquarters. We inferred the probability of symptom onset per day since infection based on the density distribution of the incubation period to stratify the serial interval distribution in Period 1 (20 January–10 February 2020) and Period 2 (25 July–4 December 2021), without and with expanded testing or implementation of social distancing strategies, respectively. Assuming both no correlation as well as positive and negative correlations between the incubation period and the serial interval, we estimated the proportion of pre-symptomatic transmission in South Korea as 43.5% (accounting for correlation, range: 9.9–45.4%) and 60.0% (56.2–64.1%) without and with expanded testing, respectively, during the Delta variant’s predominance. This study highlights the importance of considering pre-symptomatic transmission for COVID-19 containment and mitigation strategies because pre-symptomatic transmission may play a key role in the epidemiology of COVID-19.

## 1. Introduction

The coronavirus disease (COVID-19) was first reported in Wuhan, Hubei Province, People’s Republic of China, in December 2019, and the severe acute respiratory syndrome coronavirus 2 (SARS-CoV-2) has spread worldwide and has caused more than 500 million cases and 6 million deaths as of May 2022. In South Korea, COVID-19 had afflicted more than 17 million individuals and had caused more than 20,000 deaths by May 2022.

The transmission of SARS-CoV-2 from an individual prior to symptom onset—that is, pre-symptomatic transmission—is a well-documented phenomenon and ranges from 45.9% (95% confidence interval [CI] 42.9–49.0%) to 69.1% (95% CI 66.2–71.9%) globally [1]. According to the U.S. Centers for Disease Control and Prevention (CDC), 35% and 24% of all SARS-CoV-2 transmission could be attributed to pre-symptomatic individuals and those who remained asymptomatic, respectively [2]. Pre-symptomatic individuals with SARS-CoV-2 infection are highly infectious immediately before and around the time of symptom onset and appear to transmit the virus efficiently, particularly within households [3]. A study of a SARS-CoV-2 outbreak in a long-term care facility showed that the infectious virus could be isolated in cultures from the real-time reverse transcription–polymerase chain reaction (RT-PCR)-positive upper respiratory tract specimens of pre-symptomatic patients as early as 6 days prior to symptom onset [4]. The risk of transmission from pre-symptomatic individuals is reported to be less than that from symptomatic individuals [5,6,7]. However, pre-symptomatic individuals are less likely to isolate themselves from other people and, thus, the extent to which transmission from such individuals contributes to the pandemic is unknown. In addition, modeling studies have indicated that the widespread worldwide occurrence of COVID-19 would not have been possible without substantial pre-symptomatic transmission [3]. A recent mathematical modeling study demonstrated the role of asymptomatic infection in the transmission potential of COVID-19 at the population level but did not explicitly incorporate the role of pre-symptomatic transmission [8].

This study was conducted to estimate the proportion of pre-symptomatic transmission during the Delta variant’s predominance in South Korea by using individual-level data from a large number of confirmed local cases. Individual-level observations, including the timing of exposure, symptom onset, and onward transmission, are required to calculate the probability of pre-symptomatic transmission. However, due to the limited availability of individual-level data, we estimated the probability of pre-symptomatic transmission using the density distribution of the incubation period and the serial interval. Furthermore, we present our analysis of the incubation period and serial interval in accordance with the earlier and later phases of the outbreaks, given that control measures, such as expanded testing and implementation of social distancing strategies, were introduced during the time frame of the data that were analyzed.

## 2. Materials and Methods

In this retrospective population-based study, individual-level records, including age, sex, state of residence, autochthonous (local transmission)/imported case, and date of death, of confirmed COVID-19 cases were obtained from the database provided by the Korea Disease Control and Prevention Agency (KDCA) and Central Disease Control Headquarters. Some of these records included information on the date of contact with an infector and date of symptom onset and reporting. Specifically, data from 279,059 cases, recorded between 25 July 2021 and 4 December 2021 during the Delta variant’s predominance in South Korea, were extracted from the database and used to identify case reports that included information on contact tracing with matched pairs (i.e., case numbers of infectors or infectees) and dates of symptom onset. The dataset was filtered to obtain the data for dates of symptom onset, and 187,936 out of 279,059 cases in Period 2 were reported to have developed symptoms; this indicates that 33% of the reported cases were asymptomatic. Furthermore, the dataset was filtered to obtain the data for case numbers of infectors or infectees. Based on these infection pairs, we estimated the serial interval and incubation period distribution, which were then used to identify the proportion of pre-symptomatic transmission that is defined as the proportion of onward transmission during the infector’s incubation period. Specifically, we approximated the probability of pre-symptomatic transmission as the fraction of samples where the serial interval is shorter than the incubation period [9]. We assume that, if the incubation period of an infector and of an infectee are taken to be independent and identically distributed, then the serial interval can be used to approximate the generation time [1].

To estimate the proportion of pre-symptomatic transmission without and with expanded testing and social distancing strategies, we considered the two study periods (Period 1: 20 January 2020 to 10 February 2020; Period 2: 25 July 2021 to 4 December 2021). In South Korea, the first confirmed case was reported on 20 January 2020, and during Period 1, 28 COVID-19 cases were reported and limited expanded testing or implementation of social distancing strategies was undertaken. Following the identification of the local transmission cluster linked to members of the Shincheonji religious group in Daegu on 20 February, testing was expanded to include suspected cases based on the physicians’ judgement and individuals with no travel history. Period 2 has been chosen to incorporate the effects of interventional strategies including expanded testing during the Delta variant’s predominance into our analysis, given that the detection rate of the Delta variant accounted for more than 50% of local cases after 25 July 2021. To reduce the change in the serial interval by virus mutation, Period 2 was limited to the duration before the first appearance of the Omicron variant—that is, until 4 December 2021.

**Figure 1:**
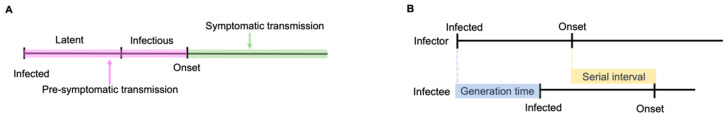
The demonstrative timeline of transmission and symptom onset. (**A**) Timeline of pre-symptomatic and symptomatic transmissions; (**B**) Graphical interpretation of the generation time and serial interval using the timeline of transmission chain for an infector and infectee pair.

For Period 1, we used the reported mean incubation period (5.5 days; 95% CI 4.0–8.1 days) and serial interval (6.5 days; 95% CI 4.3–9.7 days), both of which were estimated based on the first 28 cases in South Korea [10]. For the mean incubation period in Period 2, we used the prior estimate for the Delta variant (i.e., 4.4 days; 95% CI 3.9–5.0 days), and estimated the serial interval based on individual-level records, including information on date of contact with an infector, date of symptom onset, and date of reporting of confirmed COVID-19 cases to the KDCA and Central Disease Control Headquarters. Specifically, 187,936 COVID-19 cases were identified during Period 2 in South Korea. In the abovementioned datasets, some case reports included information pertaining to contact tracing with matched pairs (i.e., case numbers of infectors and infectees). The serial interval was defined as the time interval between symptom onset for both the infector and the infectee in the transmission chain (Figure 1). To estimate the serial intervals, we identified transmission pairs that comprised the date of symptom onset for both the infector and infectee; accordingly, 29,009 transmission pairs were identified in Period 2.

To calculate the proportion of pre-symptomatic transmission, we randomly inferred the incubation period and serial interval distributions before comparing each pair. In general, a serial interval that is shorter than the incubation period is considered indicative of pre-symptomatic transmission (Figure 1). To examine the proportion of pre-symptomatic transmission assuming no correlation between incubation period and serial interval, we randomly drew samples from the distributions and paired them randomly. By contrast, when considering positive and negative correlations, we aligned samples of the incubation period and serial interval either both in ascending order or with one in ascending and the other in descending order (rank ordering) prior to calculating the proportion of pre-symptomatic transmission, respectively [9].

## 3. Results

**Figure 2:**
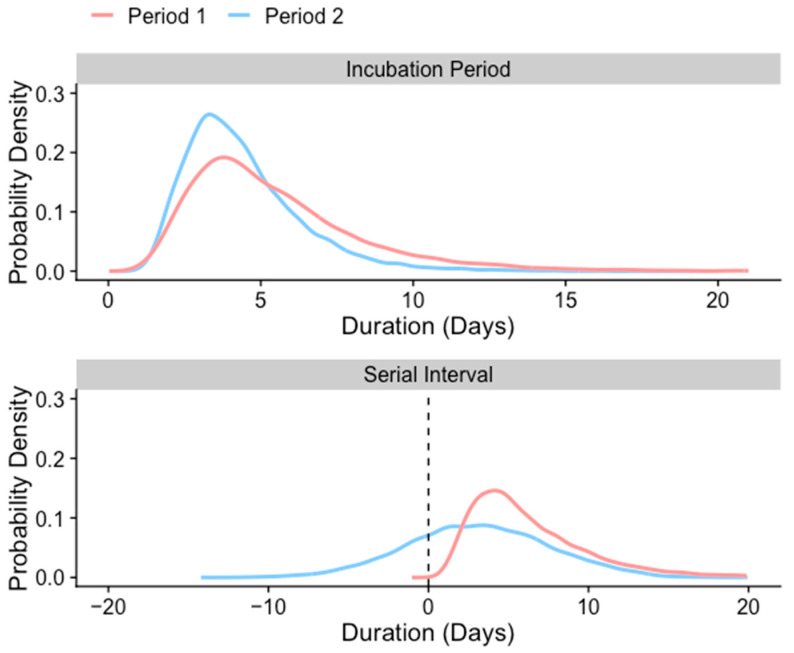
Probability density functions for the reported incubation period and serial intervals based on which the estimates were obtained in this study.

For Period 1, wherein there was no expanded testing or implementation of social distancing strategies, the probability density functions for the reported incubation period and serial interval were reported by fitting the log-normal distribution (mean 5.5 days, 95% CI 4.0–8.1 days and mean 6.5 days, 95% CI 4.3–9.7 days, respectively) from an earlier study (Figure 2) [10]. For Period 2, when the Delta variant was the predominant SARS-CoV-2 variant in South Korea, the incubation period was previously estimated by fitting the lognormal distribution (mean 4.4 days, 95% CI 3.9–5.0 days) [11], and we estimated the serial interval (mean 3.2 days, 95% CI 3.1–3.2 days) from the individual-level COVID-19 case records in South Korea (Figure 2 and Figure S1 in the Supplementary Materials). 

**Figure 3:**
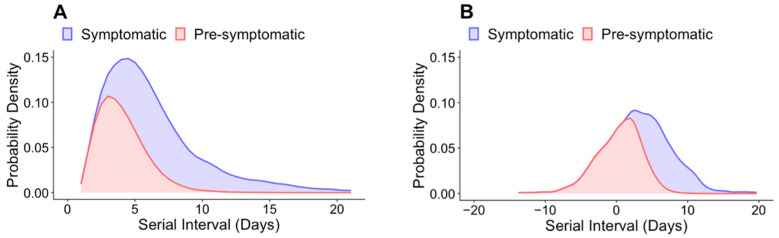
Estimated attribution of the serial interval into pre-symptomatic transmission and symptomatic transmission for (**A**) Period 1 and (**B**) Period 2. These estimates assume that there is no correlation between the incubation period and serial interval estimates.

Using these estimates for the incubation period and serial intervals, we examined the proportion of onward transmission that occurred during the pre-symptomatic period in South Korea. Assuming that the serial interval and incubation period distributions were uncorrelated, we estimated that, in the absence of expanded testing or implementation of social distancing strategies, the proportion of pre-symptomatic transmission of SARS-CoV-2 was 43.5% (range accounting for correlation: 9.9–45.4%; Figure 3A and Table 1). With the implementation of expanded case-finding efforts and subsequent isolation on symptom onset, it is anticipated that pre-symptomatic transmission would account for a greater proportion of all transmission events. In support of this hypothesis, the results of our simulation show that 60.0% of transmission events in Period 2 occurred prior to symptom onset in the primary case, assuming uncorrelated serial interval and incubation period distributions (Figure 3B and Table 1). Furthermore, in both periods, the pre-symptomatic transmission ratio gradually decreased as the serial interval increased (Figure 3). For periods 1 and 2, the proportion of symptomatic transmission was greater than 0.5 when the serial interval was at least 4.9 and 4 days, respectively (Figure 3).

**Figure 4:**
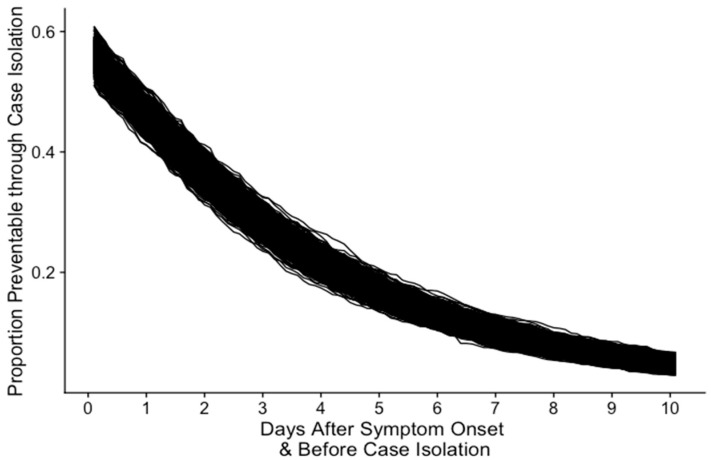
Proportion of potentially preventable onward transmission based on the time to case isolation (days) after symptom onset. These estimates are based on parameters from scenarios where there are no social distancing measures.

Depending on the time of case isolation after symptom onset, a proportion of symptomatic transmission is preventable. If the case isolation is performed immediately after symptom onset, our results show that up to 60% of symptomatic transmission could be prevented (Figure 4). However, if the case isolation is performed after approximately three days, the proportion of preventable symptomatic transmission is decreased by half and is followed by the less preventable symptomatic transmission.

## 4. Discussion

We calculated the proportion of pre-symptomatic transmission of COVID-19 cases in South Korea based on the extent of overlap in the distribution of the incubation period and the serial interval. We found differences in the estimated serial intervals between the periods wherein social distancing measures including expanded testing or implementation of social distancing strategies were present or absent. Consistent with the results of other studies [12], our results indicate that the serial interval in South Korea decreased with a relatively high level of restrictions. Moreover, our study shows that interventions, such as rapid isolation of symptomatic individuals, would result in the occurrence of a greater proportion of transmission earlier in the infectious period, leading to more pre-symptomatic transmission, which is consistent with the results of earlier studies [1,12,13].

The portion of pre-symptomatic transmission is a central quantity for infection control; however, for COVID-19, there is a substantial amount of uncertainty in the contribution of asymptomatic infections to the total transmission dynamics [14]. The possibility of pre-symptomatic transmission in COVID-19 cases is supported by the findings that both the viral genome and the live virus have been detected in upper respiratory samples prior to symptom onset [1,4,15] and that symptom onset in the infectee preceded symptom onset in the infector [16,17]. Pre-symptomatic transmission of SARS-CoV-2 in COVID-19 is further supported by quantitative studies based on the estimation of serial intervals or by similar or shorter generation times than the incubation period [17,18,19]. In addition, virus shedding peaks on or before symptom onset, and the peak transmission period is between the 2 days before and the 3 days after symptom onset, thereby indicating the transmission potential of the virus before symptom onset [20].

A prior meta-analysis that analyzed data from China, Iran, Italy, South Korea, Singapore, and Vietnam estimated that the proportion of pre-symptomatic transmission rate of COVID-19 cases ranges from 45.9% (95% CI 42.9–49.0%) to 69.1% (95% CI 66.2–71.9%), with which our estimates match (Table 2) [1]. In addition, Chun et al. used a dataset (72 transmission pairs) that was obtained from South Korea between 23 January 2020 and 31 March 2020 and estimated that 37% (95% CI 16–52%) of the transmission occurred pre-symptomatically; the authors thus concluded that the peak transmission occurred 0.72 days before symptom onset [21]. Similarly, based on data (40 transmission pairs) from several countries, Ferretti et al. inferred that 37% (95% CI 27.5–45%) of transmission was pre-symptomatic [22]. Furthermore, the analysis of the data in Beijing, China indicates that pre-symptomatic transmission may range from 15% to 81% [23]. He et al. investigated 77 transmission pairs from multiple countries and estimated that 44% (95% CI 30–57%) of transmission was pre-symptomatic [17].

Our study has some limitations. First, we approximated the generation time using the serial interval, which is the time between symptom onset in the infector and infectee, because generation time is difficult to observe directly. In our analysis, we assumed that the subtraction of the incubation period from generation time would yield the transmission time relative to symptom onset, thus facilitating an evaluation of whether transmission occurred prior to symptom onset. Second, we used population-level estimates of the serial interval and incubation period rather than individual-level estimates. Potential biases may arise from the correlation of the two estimates at the individual level, which we addressed by carrying out additional analyses under the assumption of positive or negative correlation.

## 5. Conclusions

Our study clearly indicates that substantial pre-symptomatic transmission occurs, which is consistent with the evidence reported from virological studies, case reports, and other modeling studies. Our findings highlight the need for rapid and effective case detection, contact tracing, and quarantine measures before potential symptom manifestation to ensure effective control strategies.

## Figures and Tables

**Table 1:** Overview of the scenarios tested and the corresponding estimates for the percentage of transmission that may be attributable to pre-symptomatic infections.

Scenario		Period	Serial Interval (95% CI)	Incubation Period (95% CI)	Estimated Percentage of Pre-Symptomatic Transmission If the Incubation Period and the Serial Interval Are		
					Uncorrelated	Fully Correlated	Fully Anti-Correlated
Main analysis	Without extended case finding	20 January to 10 February 2020	6.45 (4.32–9.65) [10]	5.53 (3.98–8.09) [10]	43.50%	9.90%	45.37%
	With extended case finding	25 July to 4 December 2021	3.15 (3.10–3.20)	4.4 (3.9–5.0) [11]	60.00%	64.10%	56.22%

**Table 2:** Estimates of mean proportion of pre-symptomatic transmission.

Data	Period	Number of Pairs	Mean Proportion of Pre-Symptomatic Transmission (95% CI)	Reference
South Korea	23 January to 31 March 2020	72	37% (16–52%)	[21]
Multiple Countries	-	40	37% (27.5–45%)	[22]
Beijing, China	1 January to 29 February 2020	-	- (15–81%)	[23]
Guangzhou, China	21 January to 14 February 2020	77	44% (30–57%)	[17]

## Data Availability

Not applicable.

## Supplementary Materials

The following supporting information can be downloaded at: https://www.mdpi.com/article/10.3390/jcm11143925/s1. Figure S1: Histogram and the distribution of serial intervals in Period 2. Red line shows the fitted probability density function. Table S1: The fitted probability density functions, with the estimated mean, variance, and other parameters of the probability density functions. Pseudo-code for estimating the proportion of pre-symptomatic transmission.
