# Supplementary material for: Proportion of Pre-Symptomatic Transmission Events Associated with COVID-19 in South Korea
Source: J Clin Med. 2022 Jul 6;11(14):3925. doi: 10.3390/jcm11143925 (PMC9324033; doi:10.3390/jcm11143925)
Supplement: Supplementary file 1 [file jcm-11-03925-s001.zip › jcm-1761147-supplementary.pdf]

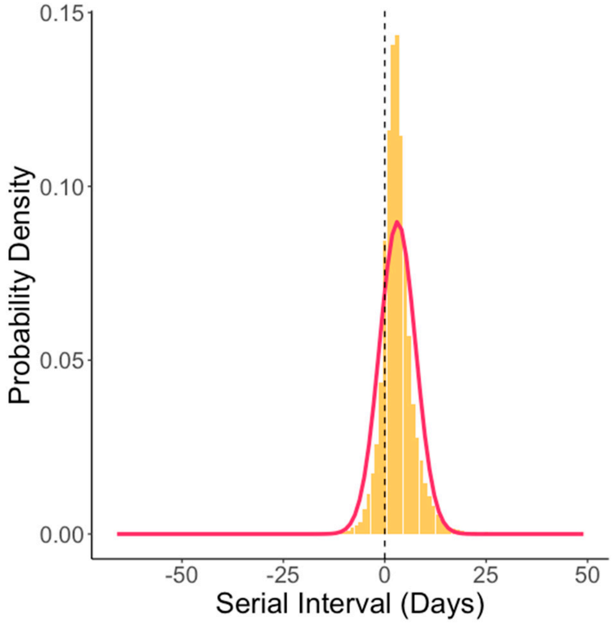

Figure S1. Histogram and the distribution of serial intervals in Period 2. Red line shows the fitted probability density function.

Table S1. The fitted probability density functions, with the estimated mean, variance, and other parameters of the probability density functions.

Period	Distribution	Mean (days)	Standard deviation (days)	Model	p_1 (95% CI)	p_2 (95% CI)	Reference
Period 1	Serial interval	6.36	0.59	Log-normal	6.45 (4.32-9.65)	4.16 (1.67-8.87)	[1]
	Incubation period	5.53	0.50		5.53 (3.98-8.09)	2.96 (1.28-6.09)	
Period 2	Serial interval	3.15	4.44	Normal	3.15 (3.10-3.20)	4.44 (4.41-4.48)	-
	Incubation period	4.38	0.42	Log-normal	4.4 (3.9-5.0)	1.9	[2]

The parameters p_1 and p_2 for the fitted PDFs normal and log-normal are expressed in the form $normal(x|p_1, p_2) = normal(\mu, \sigma)$ and $lognormal(x|p_1, p_2) = lognormal(\mu, \sigma)$, respectively.

Pseudo code for estimating the proportion of pre-symptomatic transmission in Period 1, assuming no correlation

```
n <- 10000 # initialize the number of samples
m <- 1000 # initialize index

Randomly draw n samples from the reported distribution of serial interval to produce S
Randomly draw n samples from the reported distribution of incubation period to produce I

for j=1 to m Do
        Randomly sample S with replacement to produce a
        Randomly sample I with replacement to produce b

        for x=1 to m Do
                tmp1    <-    a[x]
                tmp2    <-    b[x]
                point[x] <- "pre-symptomatic" if tmp2 > tmp1; "symptomatic" otherwise
                random_diff[[j]][[x]] <- tmp1 - tmp2
         end for

         Y[j] <- proportion of pre-symptomatic transmission

end for

Output: mean of Y
```

Pseudo code for estimating the proportion of pre-symptomatic transmission in Period 2, assuming no correlation

```
n <- 10000 # initialize the number of samples
m <- 1000 # initialize index

Perform the normal distribution fit to empirical serial interval distributions in Period 2
Store its mean and standard deviation from the fitted model

Randomly draw n samples from the fitted distribution of serial interval to produce S
Randomly draw n samples from the reported distribution of incubation period to produce I

for j=1 to m Do
        Randomly sample S with replacement to produce a
        Randomly sample I with replacement to produce b

        for x=1 to m Do
                tmp1    <-    a[x]
                tmp2    <-    b[x]
                point[x] <- "pre-symptomatic" if tmp2 > tmp1; "symptomatic" otherwise
                random_diff[[j]][[x]] <- tmp1 - tmp2
         end for

          Y[j] <- proportion of pre-symptomatic transmission

end for

Output: mean of Y
```

Remark: For the scenario with fully correlated incubation period and serial interval, align S and I in ascending order before storing their values in a and b. For the scenario with fully correlated incubation period and serial interval, align S in ascending order and I in descending order before storing their values in a and b.

Reference

1. Won, Y.S.; Kim, J.-H.; Ahn, C.Y.; Lee, H. Subcritical Transmission in the Early Stage of COVID-19 in Korea. *International Journal of Environmental Research and Public Health* **2021**, *18*, 1265.
2. Zhang, M.; Xiao, J.; Deng, A.; Zhang, Y.; Zhuang, Y.; Hu, T.; Li, J.; Tu, H.; Li, B.; Zhou, Y. Transmission dynamics of an outbreak of the COVID-19 Delta variant B. 1.617. 2—Guangdong Province, China, May–June 2021. *China CDC Weekly* **2021**, *3*, 584.
